# DEFINING ‘OUTBREAK’: ADMINISTRATORS’ EXPERIENCES MANAGING COVID-19 AT US NURSING HOMES

**DOI:** 10.1093/geroni/igad104.3259

**Published:** 2023-12-21

**Authors:** Joan Brazier, Amy Meehan, Courtney Hawes, Renee Shield, Emily Gabois

**Affiliations:** Brown University School of Public Health, Providence, Rhode Island, United States; Brown University School of Public Health, Providence, Rhode Island, United States; Brown University School of Public Health, Providence, Rhode Island, United States; Brown University School of Public Health, Providence, Rhode Island, United States; Brown University School of Public Health, Providence, Rhode Island, United States

## Abstract

The CDC defines a COVID-19 outbreak as starting with a single positive case of a staff member or resident in a facility and ending after there have been no new cases for 14 days. According to a report from the U.S. Government Accountability Office, 94% of nursing homes in the U.S. experienced multiple COVID-19 outbreaks, resulting in a reported 1.66 million residents and 1.64 million staff members with confirmed COVID-19 as of July 2023. Research has shown that COVID-19 outbreaks froze new admissions and caused significant financial strain. This qualitative study aimed to examine how COVID-19 outbreaks were managed in U.S. nursing homes. 156 interviews were conducted with nursing home administrators from 40 facilities across the U.S. between July 2020-December 2021. Two major themes emerged from thematic analysis: 1) the definition of “outbreak” as a single individual testing positive with COVID-19 was inconsistently communicated to nursing home administrators at the start of the pandemic; and 2) administrator self-reports of COVID-19 outbreaks at their facilities indicated that many endured multiple outbreaks which were long-lasting (greater than 1 month). As policy makers and industry experts review policies around managing nursing home viral outbreaks, they should consider the emerging infection control strategies from the pandemic which successfully prevented the spread of COVID-19 and update the response to an ‘outbreak’ such that infection control mitigation measures are effective without causing restrictions that are detrimental to resident well being or nursing home financial hardship.

